# Identification by MicroRNA Analysis of Environmental Risk Factors Bearing Pathogenic Relevance in Non-Smoker Lung Cancer

**DOI:** 10.3390/jpm11070666

**Published:** 2021-07-15

**Authors:** Alberto Izzotti, Gabriela Coronel Vargas, Alessandra Pulliero, Simona Coco, Cristina Colarossi, Giuseppina Blanco, Antonella Agodi, Martina Barchitta, Andrea Maugeri, CT-ME-EN Cancer Registry Workers, Gea Oliveri Conti, Margherita Ferrante, Salvatore Sciacca

**Affiliations:** 1 Department of Experimental Medicine, University of Genoa, 16132 Genoa, Italy; izzotti@unige.it; 2 UOC Mutagenesis and Cancer Prevention, IRCCS San Martino Hospital, 16132 Genova, Italy; 3 Department of Health Sciences, University of Genoa, 16132 Genoa, Italy; gabrielafernanda.coronelvargas@edu.unige.it (G.C.V.); alessandra.pulliero@unige.it (A.P.); 4 Lung Cancer Unit, IRCCS Ospedale Policlinico San Martino, 16132 Genova, Italy; simona.coco@hsanmartino.it; 5 Mediterranean Oncological Institute (IOM), 95029 Catania, Italy; cristina.colarossi@grupposamed.com (C.C.); giusi.blanco@grupposamed.com (G.B.); sciacca@unict.it (S.S.); 6 Department of Medical and Surgical Sciences and Advanced Technologies “G.F. Ingrassia”, University of Catania, 95123 Catania, Italy; agodia@unict.it (A.A.); martina.barchitta@unict.it (M.B.); andrea.maugeri@unict.it (A.M.); marfer@unict.it (M.F.); 7 Catania, Messina, Enna Cancer Registry, Via S. Sofia 87, 95123 Catania, Italy; segreteria.rti@policlinico.unict.it

**Keywords:** no-smokers lung cancer, microRNA, DNA adducts, environmental risk factors

## Abstract

MicroRNA and DNA adduct biomarkers may be used to identify the contribution of environmental pollution to some types of cancers. The aim of this study was to use integrated DNA adducts and microRNAs analyses to study retrospectively the contribution of exposures to environmental carcinogens to lung cancer in 64 non-smokers living in Sicily and Catania city near to the Etna volcano. MicroRNAs were extracted from cancer lung biopsies, and from the surrounding lung normal tissue. The expression of 2549 human microRNAs was analyzed by microarray. Benzo(a)Pyrene-DNA adducts levels were analyzed in the patients’ blood by HPLC−fluorescence detection. Correlations between tetrols and environmental exposures were calculated using Pearson coefficients and regression variable plots. Compared with the healthy tissue, 273 microRNAs were downregulated in lung cancer. Tetrols levels were inversely related both with the distance from Etna and years since smoking cessation, but they were not significantly correlated to environmental exposures. The analysis of the microRNA environmental signatures indicates the contribution of environmental factors to the analyzed lung cancers in the following decreasing rank: (a) car traffic, (b) passive smoke, (c) radon, and (d) volcano ashes. These results provide evidence that microRNA analysis can be used to retrospectively investigate the contribution of environmental factors in human lung cancer occurring in non-smokers.

## 1. Introduction

The lung epithelium undergoes a series of morphological changes before becoming invasive, such as hyperplasia, metaplasia, and finally dysplasia and in situ carcinoma. The two main types of lung cancer are small and non-small cell lung cancer (NSCLC), accounting for 80% to 85% of all cases. The three most common histological forms of NSCLC are epidermoid or squamous cell carcinoma, large cell carcinoma, and adenoma; among them, adenocarcinoma accounts for 40% of all lung cancer cases. [1] Environmental factors, including smoking, diet, and environmental carcinogens, are important in the pathogenesis of cancers through epigenetic modifications.

Interestingly, some miRNAs are dysregulated in NSCLC, which may be indicative of disease status or therapeutic outcome. [2]. It is nowadays well established that environmental pollutants alter the microRNA machinery, a situation resulting in adaptive effects in the case of short-term exposures and adverse effects in the case of long-term exposure [3]. MicroRNAs (miRNAs) are little non-coding RNA molecules that have different regulatory roles in cell differentiation, proliferation, and survival. miRNAs can inhibit complementary mRNA targets, regulating translation through RNA degradation, and are found to be dysregulated in numerous diseases, including cancer, frequently altered owing to mutations or transcriptional changes of the enzymes that regulate miRNA biogenesis [4]. However, approximately 25% of lung cancer cases worldwide, mainly adenocarcinoma, cannot be attributed to tobacco smoking; lung cancer in never smokers is the seventh leading cause of cancer deaths worldwide [5]. According to clinical experience, a different epidemiology and natural history are observed between lung cancers in never smokers and those in smokers [6], suggesting that lung cancer in never smokers is a “different” disease, with a specific etiology and molecular differences. A still unsolved problem is the evaluation of the environmental contribution to lung cancer in non-smokers. Concerning the use of miRNAs as biomarkers for lung cancer, it has been noticed that miR-146a-5p, miR-324-5p, miR-223-3p, and miR-223-5p may regulate cancer-associated gene expression, as they are down expressed in the normal bronchial airways of smokers with lung cancer [7]. Our previous research addresses the identification of a reliable cluster of miRNAs to be used as early cancer predictors, considering the high heterogeneity of lung cancer patients. Differences between miRNA profiles based on gender have being suggested in animal models of adenoma-free and adenoma bearing mice exposed to mainstream cigarette smoking [8]. Moreover, the early diagnosis of lung cancers using miR-33a-5p and miR-128-3p signatures has being proposed, as they are linked to tumor suppression processes. [9] Different response to air pollutants as particulate matter, ultrafine particles, nitrogen oxides, black carbon, and carbon oxides (CO and CO_2_) may be related to a different expression of miR-92a-3p, miR-484, and miR-186-5p, linking traffic-related exposure to disease risk [10]. It is known that microRNA and other molecular alterations (oncogene mutations, DNA adducts, transcriptional silencing activation, and proteosome alteration) induced by environmental pollution are quite specific, as each pollutant preferentially alters the expression of a cluster of identifiable molecular fingerprints [11]. This issue has been explored in a peculiar environmental situation characterized by the presence of an active volcano (Etna) near to the analyzed population (Sicily, Italy). Indeed, volcanic dust from Etna has been related to a higher risk of pleural mesothelioma, thyroid cancer [12], and other non-malignant respiratory diseases [13], as well as having a possible pathogenic role in the epidemiology of amyotrophic lateral sclerosis [14] and neurodegenerative diseases [15]. Etna’s volcanic dust is also a vector of atmospheric pollutants, such as polycyclic aromatic hydrocarbons and particulates rich on mercury [16]. Furthermore, in a recent study, the surface reactivity of ash from Etna’s activity was characterized and, although most of the released elements are below the Italian legal limits, a few inorganic elements (B, Cd, Ni, and As) are released in a higher level than permitted, with possible negative consequence for human health [17]. miR-19a, miR-30e, miR-335, and miR451a in peripheral blood have been suggested as potential biomarkers of radon radiation damage [18]. To the best of our knowledge, the correlation between volcanic ash exposure and miRNA alterations has never been explored.

To ascertain whether or not there was an association between the cancer-related pattern of microRNA alteration induced by passive smoke exposure, airborne car traffic pollution, volcano ash, and radon exposure, we herein present a retrospective study to investigate the correlation between miRNAs expression and DNA adducts in lung cancer tissue and healthy tissue in non- and former-smokers in order to shed light on the differential contribution of environmental factors to the lung carcinogenesis process. The aim of this paper is to use integrated DNA adducts and miRNA analyses in order to shed light on the differential contribution of environmental factors to lung carcinogenesis in non-smokers.

The presented approach integrates both molecular biomarkers (DNA adducts) and post-transcriptional regulation analysis (miRNAs expression in lung tissue) in order to shed light on the differential contribution of environmental factors to lung carcinogenesis in non- and former-smokers, ranking each environmental risk factor, mainly including passive smoke, car traffic pollution, volcano ashes, and radon. The presented approach can contribute to prioritize public health intervention for the primary prevention of lung cancer in non-smokers.

## 2. Materials and Methods

### 2.1. Patient’s Recruitment and Sampling

Patient recruitment was carried out in four hospitals of Catania (University Hospital “G. Rodolico, San Marco”; “Garibaldi-Nesima” Hospital; “Cannizzaro” Hospital; and “Morgagni” Clinic) and “San Vincenzo” Hospital of Taormina (Messina province). The study protocol was performed according to the Declaration of Helsinki and was approved by the Ethic Committee Catania 1 (n. 11,778 released on 17 March 2015) and Ethic Committee Catania 2 (346/C.E. released on 28 May 2015), respectively.

**Figure 1:**
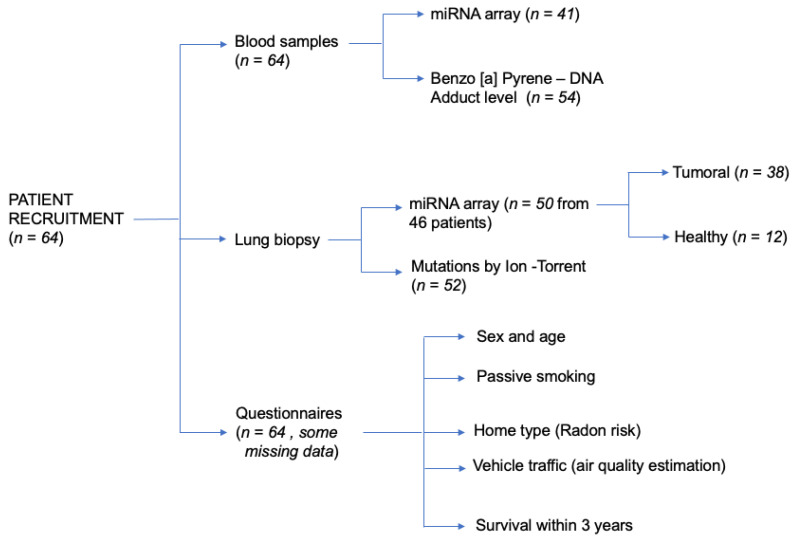
Patients enrolled-characteristics and sample sizes of analytical determinations carried out.

The criteria used for the patient enrolment was as follows: over 18 years of age, have lung cancer for which surgery treatment has been indicated, have been non-smokers or former smokers for at least 5 years, and have signed the written informed consent. No restriction was made regarding the sex of patients or the morphology of the reported neoplastic lesions. Both the neoplastic and healthy tissue samples were taken from the same patient and the tissue samples were obtained directly from the pathological anatomies of the hospitals involved in the project. Instead, the blood samples were collected by the thoracic surgery units of the hospitals. The interviews were carried out directly in the thoracic surgery wards by the cancer registry doctors involved in the study. A total of 64 patients were finally enrolled. All of the patients lived near the Etna volcano (average 56 km away, min 13 km away, and max 152 km away), their average age was 69.02 years old (min 43 years old and max 84 years old), 34.4% were female, and 20.3% of patients died within three years after the biopsy. A total of 15 subjects had never smoked, while 20 were former smokers for more than 20 years, 13 for 11 to 19 years, and 9 for 10 to 5 years, and the smoking habits data was missed for 14 patients. Data were collected by trained personnel using a semi-structured questionnaire to obtain information on the sociodemographic and lifestyle data, including smoke history, nutrition, home characteristic, and home location (for Radon and urban traffic pollution exposure evaluation) (Figure 1).

### 2.2. Lung Biopsies Collection

Lung biopsy specimens (*n* = 64) were collected at the onset of disease from patients who were diagnosed with lung cancer between 2015 and 2018, and were referred to the Catania, Messina, Enna Cancer Registry, Italy. The study was approved by Ethics committee—informed consent was obtained by “G. Rodolico—San Marco” University Hospital. All patients were classified as cases according to the 2021 ICD-10-CM Diagnosis Code C34.90. microRNA were comparatively evaluated in the cancer and surrounding normal tissue, as identified by intra-surgery histopathological analysis.

### 2.3. miRNA Extraction

The total RNA was extracted from the lung biopsies using a standardized protocol that combined a phenol/guanidine-based lysis of samples and silica-membrane-based purification. In brief, 30 mg of the starting material was first disrupted and homogenized in 700 µL of the QIAzol Lysis Reagent, using the TissueRuptor II (Qiagen, Milan, Italy) for 20–40 s. Next, the total RNA was purified from the homogenate using the miRNeasy Mini Kit (Qiagen, Milan, Italy), as described by the manufacturer’s protocol. Purification of RNA was automated on the QIAcube instrument (Qiagen, Milan, Italy).

### 2.4. miRNA-Microarray and Bioinformatic Analyses

The miRNA expression profiling was carried out with the Agilent platform following the miRNA Microarray protocol v.3.1.1 (Agilent Technologies, Santa Clara, CA, USA). Briefly, 50 ng of total RNA, containing miRNAs and Spike-in controls underwent dephosphorylation and a labelling step with Cyanine 3-pCp. The Cy3-labeled RNA was then purified using a Micro Bio-Spin P-6 Gel Column (Bio-Rad Laboratories, Inc., Hercules, CA, USA) and hybridized on Human miRNA microarray slide 8 × 60 K (Agilent Technologies; including 2549 miRNAs, miRBase 21.0) at 55 °C for 20 h. After washing, the slides were scanned with a G2565CA scanner (Agilent Technologies) and the images were extracted by Feature Extraction software v.10 (Agilent Technologies). The microarray raw data were previously deposited in the Gene Expression Omnibus (http://www.ncbi.nlm.nih.gov/geo/; accessed on 8 March 2021, GEO accession number GSE169587, ID: 200169587) for a previous study from the same authors [4].

The bioinformatic analyses of the microarray data were performed with GeneSpring software (GeneSpring Multi-Omic Analysis version 14.9 Build 11,939 by Agilent Technologies). For each specimen, the intensities of the replicated spots on each array were log transformed and averaged. All the lung-tissue-miRNA raw data files from the Agilent Technologies Microarray Scanner System G2565CA were imported to GeneSpring using miRNA analysis type, Technology 70156_v21_0, without baseline transformation. Data processing was performed by 3D principal component analysis (PCA) scores and Hierarchical Clustering.

Comparisons between sets of data were done by evaluating the fold variations. A volcano plot *t*-test analysis for all the miRNA entities between the averaged tumoral and healthy tissues was run, using fold change ≥2 and *p*-value ≤ 0.05, without multiple test corrections as the threshold values.

miRNAs related to five different environmental exposures (Environmental Exposure miRNA Signature) were determined analysing lung cancer related miRNAs comparatively in exposed vs. non-exposed subjects. Environmental exposures were determined for each patient using the questionnaires information according to (a) passive smoking at home, (b) passive smoking at work, (c) vehicle traffic at home, (d) distance (km) from the Etna volcano, and (e) radon risk according to home type.

To understand the relationship between environmental exposure signatures and their biological significance in lung cancer tissues, a target detection for each environmental exposure signature was done using the TargetScan prediction database. This database was chosen as it is the most updated database, and the number of target genes are reported for different cut-offs. The most interesting genes targeted by environmental exposure miRNA signatures that are potentially related with each environmental exposure were identified.

After the target detection, a prediction model was build using a Neural Network class prediction algorithm for each Environmental Exposure miRNA Signature to test the overall accuracy prediction for the chosen miRNAs.

### 2.5. Validation of Microaarray Results by qPCR Analysis

Microarray results for let-7a and miR-15 were further validated by qPCR on a subset of 20 patients for which enough RNA was still available. These miRNAs were selected because of their relevance lung carcinogenesis. The total RNA (10 ng) was reverse transcribed using miR-specific stem-loop RT primers (TaqMan MicroRNA Assays; Applied Biosystems, Thermo-Fisher) and components of the High Capacity cDNA Reverse Transcription kit (Life Technologies) according to the manufacturer’s protocols.

The expression levels of individual miRNAs were detected by subsequent RQ-PCR using TaqMan MicroRNA assays (Life Technologies) and a Rotor Gene 3000 PCR System Corbett (Qiagen) using standard thermal cycling conditions in accordance with manufacturer recommendations. The PCR reactions were performed in triplicate in final volumes of 30 µL, including inter-assay controls (IAC) in order to account for variations between runs. RT-PCR (TaqMan MicroRNA Assays; Applied Biosystems, Thermo-Fisher) was used to quantify the expression of let-7a and miR-15 according to the manufacturer’s instructions. To normalize the data for quantifying miRNAs, the universal small nuclear RNU38B (RNU38B Assay ID 001004; Applied Biosystems) as an endogenous control was used.

### 2.6. Benzo[a]Pyrene-DNA Adduct Levels in Human White Blood Cells

Hydrolyzed BPDE adducts or Tetrol I-1 and Tetrol II-2 were analyzed in lymphocyte DNA through the modified High-Performance Liquid Chromatography–Fluorescence (HPLC−FL) method described by Alexandrov et al. [19] and Oliveri Conti et al. [20].

Briefly, lymphocytes were separated from whole blood samples using HISTOPAQUE-1077 (Sigma-Aldrich Chemie Gmbh, Munich, Germany). The lymphocyte DNA was extracted using the DNeasy Blood and Tissue kit according to customer’s procedure (Qiagen, Milan, Italy).

Hence, DNA was subjected to a procedure of hydrolysis and purification, and Tetrols were quantified according to the methodology of Oliveri Conti et al. [20]. HCl, also if hypergrade certified, can contain traces of fluorescent active contaminants that could interfere with the peak detection of the studied analytes and reduce analytical sensitivity of the method.

To avoid this important bias in the sample preparative phase, all of the HCl impurities visibly reactive to the FL detector were deleted by chemical purification [20]. To improve the sensibility of detection, Thermo Scientific™ PEEK Capillary Tubing (0.005 in) was used. The extracted and purified DNA was dissolved in 1 mL of water and analyzed in a Varian Pro Star System HPLC using a TOSOH (C18 RP 25 × 0.46 cm, 5 µm) column with the following elution program: 15 min with 20% water/acetonitrile of equilibrium phase, 5 min with 20% water/acetonitrile and 60 min to acetonitrile (100%) (slop of 1) and, finally 10 min to 100% acetonitrile.

An isocratic program (0.85 mL/min) was used, and the FL detector (FLD) was programmed to 344 nm (ext.) and 388 nm (em.) for the excitation and emission wavelengths, respectively. The sensitivity of the FLD was fixed to a high modality. The wavelength of UV−VIS detector (UV) was set at 238 nm, permitting the dual detection of both Tetrols (I-1 and II-2).

The chromatographic system was calibrated using external certified pure standards of Tetrol I-1 and Tetrol II-2 (purity 99.0%) (Chemical Carcinogen Reference Standard Repository, Kansas City, MO, USA).

Recoveries were 94% and 82% for Tetrol I-1 and Tetrol II-2, respectively. The processing of reagent blank disclosed no trace of Tetrol I-1 and Tetrol II-2. The linearities (*R^2^*) obtained of FLD were 0.9980 and 0.9990 for Tetrol I-1 and Tetrol II-2, respectively. For UV, the *Rs^2^* were 0.9850 e 0.9803 for Tetrol I-1 and Tetrol II-2, respectively. MDL were 2.0 pg/mL and 3.1 pg/mL for Tetrol I-1 and Tetrol II-2, respectively. The validated method permitted detecting Tetrol I-1 and Tetrol II-2 in a minimum of 3µg of extracted DNA.

### 2.7. Statistical Analysis

The statistical significance of the differences between groups was evaluated by ANOVA, followed by Student’s *t*-test for unpaired data. *p*-values lower than 0.05 were regarded as statistically significant. Correlations (i.e., Pearson coefficients and regression variable plots) between Benzo[a]Pyrene-DNA adducts and the different environmental exposures were calculated with IBM SPSS statistics (Version 22).

## 3. Results

### 3.1. Comparison of miRNA Profile between Healthy and Cancer Tissue in Lung

**Figure 2:**
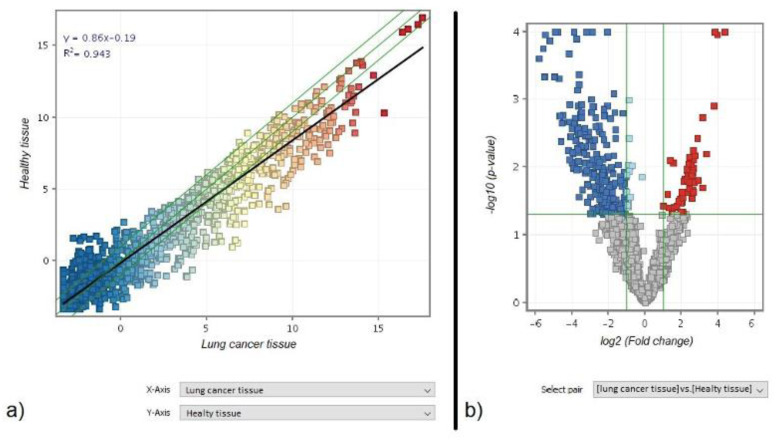
(**a**) Scatter plot analysis comparing miRNA expression (dots) according to their level of expression in healthy (vertical axis) vs. cancer (horizontal axis) tissues of the examined patients. The miRNA colour reflects the signal intensity (red is high, yellow is intermediate, and blue is low). The diagonal green lines indicate the two-fold variation interval. The best-fit regression line is reported in black. Its slope towards the horizontal axis reflects the overall downregulation of miRNA expression in cancer compared with healthy lung tissue. (**b**) Volcano plot analysis identifying miRNAs with an altered expression above two-fold (horizontal axis) and above the statistical significance threshold (*p* < 0.05) (vertical axis) in cancer vs. healthy lung tissue, either downregulated (blue) or upregulated (red).

The scatter plot analysis of the miRNA-arrays comparing healthy and lung cancer tissues presents a general trend toward down regulation in cancer tissue, as indicated by the slope of the black regression line (Figure 2a). The volcano plot analysis highlighted a list of 273 miRNAs that were altered more than two folds and above the statistical significantly threshold (*p* < 0.05) in cancer vs. healthy tissue. Of these miRNAs, 222 were down-regulated (blue dots) and 51 were up-regulated (red dots) (Figure 2b). This group represents the Lung Cancer Related miRNAs. This list is reported in the supplementary material (Supplementary Table S1) and includes well established oncogenic miRNAs, such as an extensive downregulation of the whole let-7 miRNA family and of the miR-34 family, an established effector of p53.

**Figure 3:**
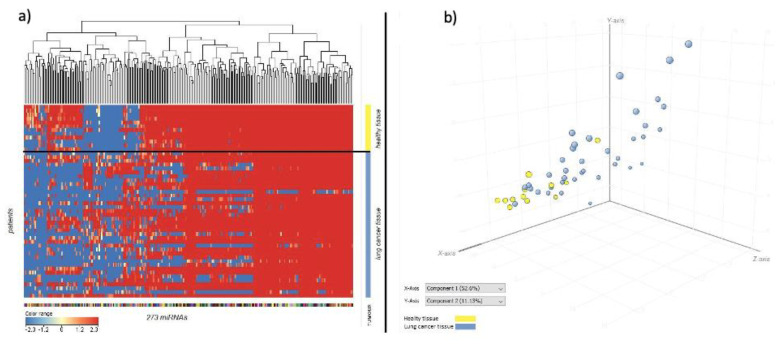
(**a**) Clustering hierarchical analysis reporting the expression of the 273 lung cancer related miRNAs in healthy tissue (vertical axis, yellow bar) and lung cancer tissue (vertical axis, blue bar) in the 50 samples tested (horizontal lines). (**b**) Principal component analysis of variance identifying the samples from healthy tissues (yellow dots) and cancer tissues (blue dots) according to the variance of the expression of the 273 lung cancer related miRNAs. dots size = principal component analysis score.

The lung cancer related miRNAs downregulation trend was well distinguishable between the cancer and healthy tissue, as also indicated by the hierarchical cluster analysis (Figure 3a), where healthy tissue profiles (yellow bar) were clustered in the upper part of the hierarchical tree separately from cancer tissue profiles (blue bar). Colour range indicates lung cancer related miRNAs’ intensity signal.

In the principal component analysis of variance (Figure 3b), healthy tissue samples (yellow dots) were mainly clustered in the lower left part of the 3D space. Instead, cancer tissue samples (blue dots) were mainly located in the upper right part of the 3D space.

The most significant predicted target genes for each of the lung cancer related miRNAs were identified using the TOP-Go Bioconductor R package and REVIGO online tool. The tree map of the most representative biological processes for lung cancer related miRNAs is reported in (Supplementary Figure S1). The most representative biological processes were as follows: regulation of RNA splicing, tissue migration, monosaccaride transmembrane transport, protein modification by small protein conjugation or removal, and cellular protein-containing complex assembly.

**Figure 4:**
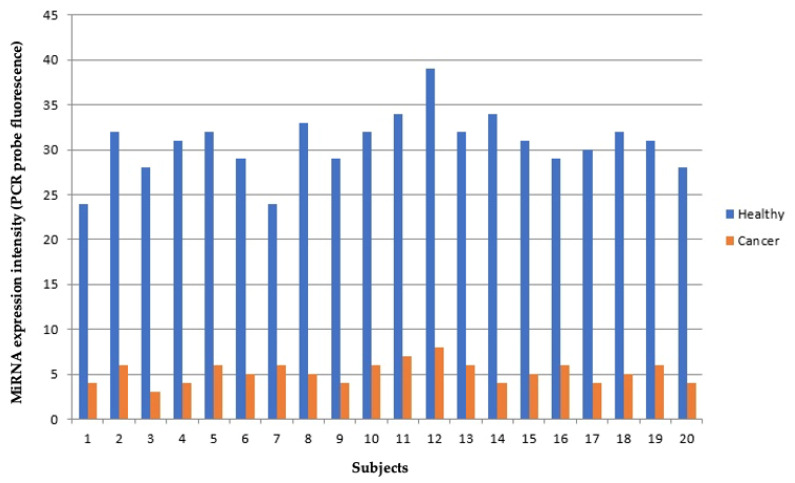
Let-7a expression in healthy (blue) vs. cancer (red) lung tissue as evaluated by qPCR in 20 patients (*x*-axis). miRNA expression intensity is expressed in fluorescent units (*y*-axis).

qPCR analyses performed for let-7a confirmed the downregulation of this miRNAs in cancer vs. healthy lung tissues of the same patient (Figure 4).

On an average, Let-7a expression was down-regulated in cancer vs. healthy tissues by 6.2 ± 1.4 fold as evaluated by qPCR, and 7.4 ± 2.2 fold as evaluated by microarray.

### 3.2. miRNA Profile Was Related with Cancer Histotype

**Figure 5:**
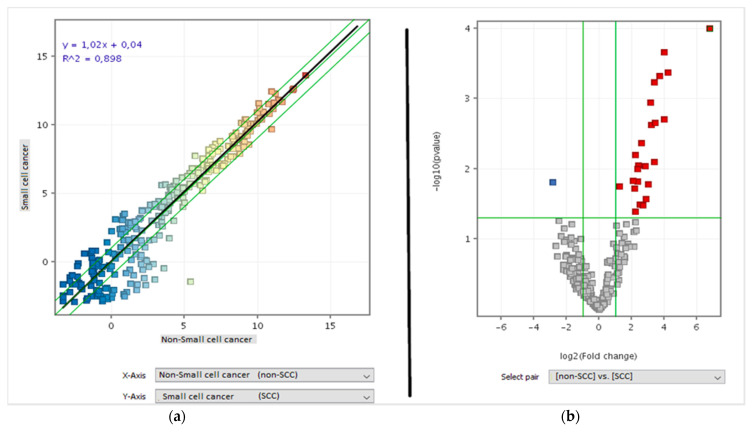
(**a**) Scatter plot analysis comparing miRNA expressions (dots) according to their level of expression in SCLC (vertical axis) vs. NSCLC (horizontal axis). miRNA color reflects the level of expression (red is high, yellow is intermediate, and blue is low). The diagonal lines indicate the two-fold variation interval. (**b**) Volcano plot analysis identifying miRNAs whose expression was altered more than two-fold (horizontal axis) and above the statistical significance threshold (*p* < 0.05) (vertical axis) in SCLC vs. NSCLC cancer downregulated (blue) or upregulated (red).

The miRNA expression was different between small cell lung cancer (SCLC) and NSCLC, as shown by the scatter plot analysis (Figure 5a). The volcano plot analysis indicated that 26 out of the 273 cancer related miRNAs were differentially expressed between SCLC and NSCL (Figure 5b). Of these miRNAs, 25 were up-regulated in NSCLC compared with SCLC and one was down-regulated. The identity of these 26 miRNAs permitted distinguishing between these two main cancer histotypes, and is reported in the supplementary material (Supplementary Table S2).

### 3.3. B(a)P-DNA Adducts and Environmental Exposures

**Figure 6:**
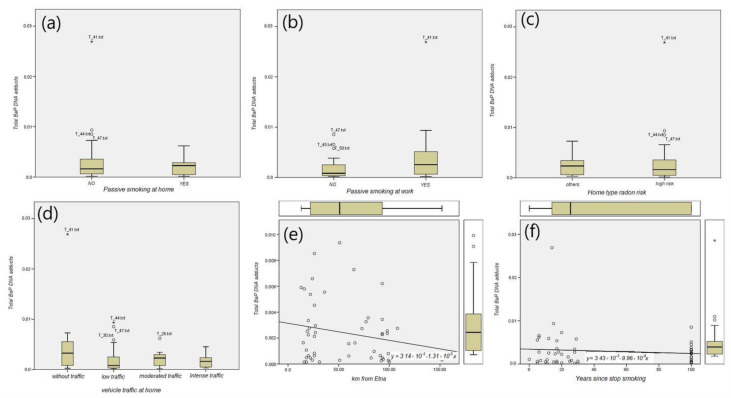
Box plot analysis for total BaP DNA adducts in: (**a**) passive smoking at home, (**b**) passive smoking at work, (**c**) radon risk home type, (**d**) vehicle traffic at home, and linear regression for (**e**) distance from the Etna volcano, and (**f**) years since smoking cessation.

The ANOVA analysis did not detect a statistically significant difference between the B(a)P-DNA adducts levels under different environmental exposures, including passive smoking at home, passive smoking at work, radon risk related to home type, and vehicle traffic at home (Figure 6). The linear regression analysis showed that the level of B(a)P-DNA adducts in the lymphocytes was inversely related with the distance from the Etna volcano (Figure 6e) and years since smoking cessation (Figure 6f).

### 3.4. Contribution of Environmental Exposures to Lung Carcinogenesis as Inferred from miRNA Profiling

Five miRNA signatures were obtained comparing the miRNA expression in the tumoral lung tissue between patients undergoing a low or high exposure for each one of the environmental exposures. The cancer related miRNAs included in each miRNA environmental signature were used to run a volcano plot analysis (FC ≥ 2, *p* ≤ 0.05). These environmental signatures were further integrated with the established miRNA, as linked with the specific exposure from the available literature.

**Figure 7:**
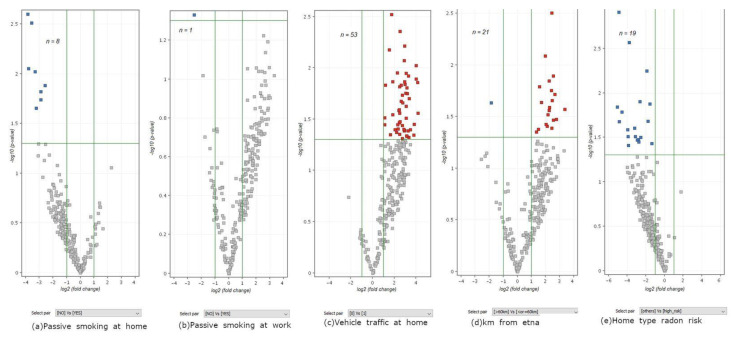
*t*-test volcano plot analyses identifying the miRNA environmental signatures (among the 273 lung cancer related miRNAs).

The number of miRNAs composing each environmental signature was as follows: (a) passive smoke at home, *n* = 8; (b) passive smoke at work, *n* = 1; (c) vehicle traffic, *n* = 53; (d), distance from the Etna volcano, *n* = 21; and (e) radon risk, *n* = 19. The volcanos plot analyses comparing miRNA expression in the lungs between unexposed vs. exposed subjects for each environmental signature are reported in Figure 7.

Accurate questionnaire data were available for 38 out of the 50 patients for whom the microRNA microarray data were collected. A volcano plot *t*-test using each EES was run to identify the altered miRNAs per patient.

**Figure 8:**
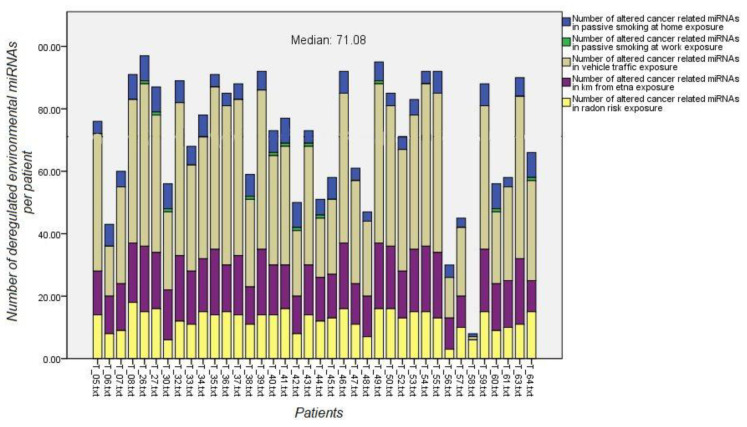
Number of altered environmental exposure miRNAs (*y*-axes, color codes) per patient (*x*-axes). Median of the summation of each environmental exposure miRNA signature are shown as horizontal colored lines. Patients having environmental risk factors above the median value underwent lung cancer contribution by this risk factor.

Environmental exposure miRNA signatures were compared by Venn diagram analysis, with the miRNAs composing the individual cancer-related signature of each patient. This approach was used to identify the relative contribution of the environmental risk factors to cancer development in each patient (Figure 8).

The number of patients with a higher number of differentially regulated miRNAs than the median value for each environmental risk factor was as follows: (a) 38 for passive smoke, (b) 38 for vehicle traffic, (c) 21 for distance from the Etna volcano, and (d) 13 for radon risk

The targeted genes for each environmental exposure signature were analyzed. The number of target genes for each signature and different *p*-values cut-off are summarized in Table 1.

Most of the genes targeted by the environmental exposure miRNA signatures with statistical significance are also expressed in the lung tissue. The gene RIF (gene reference into function) and RPKM (reads per kilobase of transcript, per million mapped reads) values in the lung tissue for each targeted gene were found using the NCBI gene database (https://www.ncbi.nlm.nih.gov/gene) accessed on 11 December 2021. These findings are reported in Table 2.

### 3.5. Evaluation of Environmental Exposure miRNA Signatures Efficacy by Neural Network Analysis

GeneSpring 14.9 was used to build a prediction model to validate the accuracy of each of the environmental exposure signatures. The network was tested in tumoral tissue. The accuracy of the environmental signature increased compared with the number of miRNAs included, as follows: (a) remarkably, for passive smoking at home, distance from the Etna volcano, and home type radon risk; (b) slightly for vehicle traffic and distance from the Etna volcano; and (c) remained equal for passive smoking at work (Table 3).

### 3.6. Environmental Exposure miRNA Signatures and B(a)P-DNA Adduct Levels

The weight of different exposures to lung cancer were profiled by analyzing the B(a)P-DNA adduct levels in the lymphocytes and miRNAs profiles in lung cancer together. For this purpose, we ranked the four exposures on the basis of the number of altered miRNAs. Correlation tests (Pearson and Spearman’s Rho) were run to evaluate whether or not B[a]P-DNA adducts were related with environmental exposure miRNA signatures. The B[a]P-DNA adduct levels were correlated only with the passive smoking miRNA signature (*p*-value = 0.049) (Table 4).

## 4. Discussion and Conclusions

Our results provide evidence that miRNAs are massively deregulated in lung cancer compared with the surrounding normal tissue. This finding is in line with other studies [21]. A major problem in using miRNA analysis for lung cancer prediction and early diagnosis is the reproducibility of the results and the invasiveness of the biopsy approach. Our cancer related miRNAs signature at least in part overlaps with the most common lung cancer miRNA-related signatures found in the literature. Indeed, 27 miRNAs were included in our cancer related signature and also in other cancer miRNA-related signatures found in literature [22]. The miRNAs downregulated in lung cancer tissue included established anti-oncogenic miRNA such as let-7, miR-30, miR-34, and miR-140. The comparison of DNA adducts and miRNA expression provided evidence that post-transcriptional alteration is massive in lung cancer, while DNA adducts alteration of an environmental origin is detectable, but only at a very low level. DNA adducts are a hallmark of the environmental contribution to the analyzed cancers, with particular reference to environmental sources of polycyclic aromatic hydrocarbons derived from combustion, such as car traffic and passive smoke. However, BaP-DNA adducts are poor predictors of cancer because (a) they can be removed by DNA repair [23], (b) the resulting mutation can be silenced thus not having phenotypical or functional consequences, and (c) the bearing cell can be removed by apoptosis. Conversely, miRNA alterations are necessary to develop lung cancer. These alterations are initially adaptive, aimed at activating defensive processes counteracting the damage induced by environmental pollutants such as DNA repair, mutation silencing, and apoptosis. However, whenever the environmental exposure lasts for decades, microRNA alterations become irreversible and commit cells to the occurrence of lung cancer environmental risk factors, inducing specific alterations in the microRNA machinery depending on the specificity of the environmental pollutant involved [3,4,5,6,7,8,9,10,11]. Accordingly, microRNA alteration is more predictive of lung cancer occurrence than genomic alterations. Indeed, microRNAs have been proposed as a tool for the early diagnosis of cancer or to identify subjects at a high risk for cancer development needing to undergo personalized cancer screening with a high frequency and sensitivity.

The negative correlation observed between the adduct level and some environmental exposures (i.e., traffic and volcano distance) is in line with the results previously published by other research groups reporting that populations undergoing long-term exposure to environmental pollution develop resistance mechanisms [24]. These events are referred to as adaptive events triggered by heterogeneous exposures [25].

The biological function of miRNAs identified as lung cancer contributors in environmental signatures reflect the pivotal role of the damage to the microRNA machinery during the carcinogenesis process. These events have been previously analyzed in detail during lung carcinogenesis in mice [8,26].

The results presented herein provide evidence that miRNA alteration in lung cancer results from exposure to environmental factors. This situation results in miRNA failure to control pivotal defensive mechanisms against cancer, mainly including oncogene suppression, cell adhesion and differentiation maintenance, cell cycle blockage, DNA and protein repair, intracellular signaling, and apoptosis.

The obtained results provide evidence that miRNA signatures may be used to identify the comparative contribution of environmental factors to lung carcinogenesis in humans. According to our environmental exposure miRNA signatures results, the contribution of the analysed environmental factors was, in decreasing order, car traffic, passive smoke, volcano, and radon. These results may be useful for stakeholders to prioritize public health intervention for the primary prevention of lung cancer in non-smokers. Indeed, it is commonly thought that the contribution of volcano ash is the main public health problem in Catania, one of the few cities in the world to be directly exposed to volcano emissions located in its near proximity. However, the obtained results indicate that the main public health problem in this town is the car traffic. Accordingly, preventive measures, such as the substitution of old with new cars characterized by low emission rates, are urgently required. In the second rank, there is passive smoke, which is a problem to be faced by stronger information campaigns and other measures (such as the increase of cigarettes price), which appears to be urgent in a country (i.e., Italy) still having the prevalence of 13 million of smokers out of a total population of 60 million.

In conclusion, the results presented herein provide more evidence that the analyses of epigenetic components may be used to face public health issues related with cancer prevention [27], with particular reference to the identification of the environmental risk factors to be prevented.

## Figures and Tables

**Table 1:** Number of predicted target genes (Targetscan database) according to each environmental exposure with a *p*-value cut off.

Environmental Exposure Signature	Number of Predicted Target Genes by *p-*Value Cut Off				
	0.05	0.01	0.005	0.001	0.0001
Passive smoking at home (n = 8)	8726	8726	8726	6	1
Passive smoking at work (n = 1)	1796	3	0	0	0
Vehicle traffic at home (n = 53)	16,132	16,132	16,132	16,132	7
Home distance from the Etna volcano (n = 21)	14,662	14,662	14,662	20	4
Home type radon risk (n = 19)	13,762	13,762	15	0	0

The most significant targeted genes for each signature are summarized in Table 2.

**Table 2:** Most significative gene target detection per environmental exposure miRNA signature by *p*-value cut-off.

Environmental Exposure Signature	*p*-Value	Genes	Gene Name
Passive smoking at home (*n* = 8)	0.001	*PTX4*	pentraxin 4
		*NAXD*	NAD(P)HX dehydratase
		*MAPK3*	mitogen-activated protein kinase 3
		*VPS16*	core subunit of CORVET and HOPS complexes
		*CACNA1S*	calcium voltage-gated channel subunit alpha1 S
		*SHARPIN*	SHANK associated RH domain interactor
Passive smoking at work (*n* = 1)	0.01	*HBG2*	hemoglobin subunit gamma 2
		*RNASE12*	ribonuclease A family member 12
		*IFT88*	intraflagellar transport 88
Vehicle traffic at home (*n* = 53)	0.0001	*LEFTY1*	left-right determination factor 1
		*RTTN*	Rotatin
		*THYN1*	thymocyte nuclear protein 1
		*CASKIN1*	CASK interacting protein 1
		*SERPING1*	serpin family G member 1
		*OGFOD2*	2-oxoglutarate and iron dependent oxygenase domain containing 2
		*PKDCC*	protein kinase domain containing, cytoplasmic
Home distance from the Etna volcano (*n* = 21)	0.001	*ARHGEF33*	Rho guanine nucleotide exchange factor 33
		*COX17*	COX17
		*RAI2*	retinoic acid induced 2
		*KIF12*	kinesin family member 12
		*COL26A1*	collagen type XXVI alpha 1 chain
		*RMDN2*	regulator of microtubule dynamics 2
		*GADD45A*	growth arrest and DNA damage inducible alpha
		*DTNA*	dystrobrevin alpha
		*HTRA4*	HtrA serine peptidase 4
		*TAS2R30*	taste 2 receptor member 30
		*STRN3*	striatin 3
		*BRINP3*	BMP/retinoic acid inducible neural specific 3
		*EYS*	eyes shut homolog
		*JAG2*	jagged canonical Notch ligand 2
		*HSD17B12*	hydroxysteroid 17-beta dehydrogenase 12
		*NIN*	Ninein
		*NAA35*	N-alpha-acetyltransferase 35, NatC auxiliary subunit
		*ZNF37A*	zinc finger protein 37°
		*GLT8D2*	glycosyltransferase 8 domain containing 2
		*DDX59*	DEAD-box helicase 59
Home type radon risk (*n* = 19)	0.005	*CNPY3*	canopy FGF signaling regulator 3
		*SUB1*	SUB1 regulator of transcription
		*CLTC*	clathrin heavy chain
		*ZNF280A*	zinc finger protein 280°
		*ALS2CR12*	(or FLACC1) flagellum associated containing coiled-coil domains 1
		*TMEM139*	transmembrane protein 139
		*BTBD3*	BTB domain containing 3
		*WDR7*	WD repeat domain 7
		*RAB15*	member RAS oncogene family
		*KRT84*	keratin 84
		*MAZ*	MYC associated zinc finger protein
		*ATAD2B*	ATPase family AAA domain containing 2B
		*PSPH*	phosphoserine phosphatase
		*PGBD1*	piggyBac transposable element derived 1
		*BEX2*	brain expressed X-linked 2

**Table 3:** Results of the neural network class prediction for each environmental exposure miRNA signature.

Environmental Exposure (Number of miRNA Entities)	Score of All miRNAs Prediction Overall Accuracy (*n* = 2570)	Environmental Exposure miRNA Signatures Prediction Overall Accuracy Score
Passive smoking at home (*n* = 8)	0.73	0.96 (+0.23)
Passive smoking at work (*n* = 1)	0.68	0.68 (+0)
Vehicle traffic at home (*n* = 53)	0.77	0.81 (+0.04)
Home distance from the Etna volcano (*n* = 21)	0.60	0.82 (+0.22)
Home type radon risk (*n* = 19)	0.83	0.96 (+0.13)

**Table 4:** Correlation between EESs miRNA and B(a)P-DNA adduct levels. (*) Statistical significance *p* < 0.05.

		Vehicle Traffic at Home	Distance from Etna Volcano	Radon Risk	Passive Smoking
Correlation Test RRSs and B[a]P-DNA adducts. Pearson (for parametric) or Spearman’s Rho (for non parametric)	Correlation Coefficient	0.54 (Parametric)	0.48 (Non-Parametric)	0.25 (Parametric)	0.34 * (Parametric)
	*p*-value	0.76	0.79	0.16	0.049

* Statistical significance *p* < 0.05.

## Data Availability

The datasets used and/or analyzed during the current study are available from the corresponding author on reasonable request.

## Supplementary Materials

The following are available online at https://www.mdpi.com/article/10.3390/jpm11070666/s1, Figure S1: Revigo TreeMap of GO-BP analysis. Most significative BPs are classified by colour with BPs subset, Table S1: *Cancer Related miRNAs*, Table S2: Significant altered miRNAs.
