# Supplementary material for: Identification by MicroRNA Analysis of Environmental Risk Factors Bearing Pathogenic Relevance in Non-Smoker Lung Cancer
Source: J Pers Med. 2021 Jul 15;11(7):666. doi: 10.3390/jpm11070666 (PMC8307636; doi:10.3390/jpm11070666)
Supplement: Supplementary file 1 [file jpm-11-00666-s001.zip › jpm-1277113-supplementary.pdf]

Table S1. ***Cancer Related miRNAs*** altered (FC>= 2, p<=0.05) in Volcano Plot Analysis between average signal in samples with non-small cell lung cancer vs. small cell lung cancer.

systematic_name	p-value	Regulation	FC
hsa-miR-1238-5p	0.011302715	up	9.62
hsa-miR-1296-5p	0.007524842	up	8.62
hsa-miR-1306-3p	0.021063296	up	8.58
hsa-miR-205-3p	4.19297E-06	up	220.64
hsa-miR-2277-3p	0.00932873	up	10.59
hsa-miR-3149	0.000521124	up	25.38
hsa-miR-326	0.019960763	down	-8.85
hsa-miR-4290	0.000451764	up	16.39
hsa-miR-4440	0.002965666	up	10.05
hsa-miR-4443	0.03872927	up	2.41
hsa-miR-4481	0.015899722	up	7.33
hsa-miR-4716-5p	0.000232035	up	20.00
hsa-miR-4763-5p	0.008582705	up	8.26
hsa-miR-4793-3p	0.002927522	up	16.70
hsa-miR-483-3p	0.002941658	up	13.22
hsa-miR-504-3p	0.002079689	up	14.95
hsa-miR-595	0.000426941	up	21.36
hsa-miR-6730-3p	0.016724579	up	6.91
hsa-miR-6743-3p	0.043577574	up	7.71
hsa-miR-6779-3p	0.016434822	up	8.81
hsa-miR-6794-3p	0.005262722	up	7.19
hsa-miR-6817-5p	0.044506542	up	7.97
hsa-miR-6826-5p	0.02230857	up	2.63
hsa-miR-6886-3p	0.014682693	up	12.86
hsa-miR-6891-3p	0.01124693	up	7.93
hsa-miR-7108-3p	0.012961295	up	6.21

Table S2. Significant altered miRNAs (Volcano plot moderated t-test, [mutated tissues] vs. [no mutated tissues], FC>=2, p<=0,05, no correction) between tumoral tissues of a total of 33 patients by Gene mutation, using only Cancer Associated miRNAs from Table 1S. TargetScan predicted if each miRNA targeted directly considered genes.

Mutation	systematic_name	p-value	Regulation	FC	mirbase accession No	Direct Target
BRAF	hsa-miR-1306-3p	0.024846	down	-32.20	MIMAT0005950	NO
BRAF	hsa-miR-139-3p	0.01998	down	-11.09	MIMAT0004552	NO
BRAF	hsa-miR-193a-5p	0.049849	down	-7.84	MIMAT0004614	NO
BRAF	hsa-miR-3620-5p	0.009772	down	-16.80	MIMAT0022967	NO
BRAF	hsa-miR-3659	0.015827	down	-35.46	MIMAT0018080	NO
BRAF	hsa-miR-521	0.00369	up	13.25	MIMAT0002854	NO
BRAF	hsa-miR-610	0.042202	down	-15.90	MIMAT0003278	NO
EGFR	hsa-miR-744-5p	0.048781	down	-6.35	MIMAT0004945	NO
KRAS	hsa-miR-106b-3p	0.009659	up	5.78	MIMAT0004672	NO
KRAS	hsa-miR-1247-5p	0.026188	up	2.56	MIMAT0005899	NO
KRAS	hsa-miR-1306-3p	0.045089	up	5.07	MIMAT0005950	NO
KRAS	hsa-miR-1537-3p	0.035001	up	4.21	MIMAT0007399	NO

KRAS	hsa-miR-15b-3p	0.048594	up	4.27	MIMAT0004586	YES
KRAS	hsa-miR-191-5p	0.041432	up	3.98	MIMAT0000440	NO
KRAS	hsa-miR-1913	0.005619	up	7.55	MIMAT0007888	NO
KRAS	hsa-miR-21-3p	0.016535	up	3.35	MIMAT0004494	YES
KRAS	hsa-miR-4440	0.027506	up	5.09	MIMAT0018958	NO
KRAS	hsa-miR-4793-3p	0.04182	up	6.36	MIMAT0019966	NO
KRAS	hsa-miR-6516-3p	0.046655	up	6.33	MIMAT0030418	NO
KRAS	hsa-miR-6804-5p	0.001817	up	12.40	MIMAT0027508	NO
KRAS	hur_5	0.033032	up	2.67	-----	------
NOTCH1	hsa-miR-3065-5p	0.02177	down	-28.11	MIMAT0015066	NO
STK11	hsa-miR-100-5p	0.044152	up	50.39	MIMAT0000098	NO
STK11	hsa-miR-106b-3p	0.027296	up	18.62	MIMAT0004672	NO
STK11	hsa-miR-144-5p	0.049938	up	73.34	MIMAT0004600	NO
STK11	hsa-miR-15b-3p	0.00585	up	44.38	MIMAT0004586	NO
STK11	hsa-miR-182-3p	0.018396	up	29.20	MIMAT0000260	NO
STK11	hsa-miR-190a-5p	0.017345	up	40.56	MIMAT0000458	NO
STK11	hsa-miR-191-5p	0.005873	up	33.11	MIMAT0000440	NO
STK11	hsa-miR-1913	0.010622	up	37.54	MIMAT0007888	NO
STK11	hsa-miR-26b-3p	0.010553	up	43.63	MIMAT0004500	NO
STK11	hsa-miR-29a-5p	0.049188	up	38.69	MIMAT0004503	NO
STK11	hsa-miR-29b-1-5p	0.011177	up	118.38	MIMAT0004514	NO
STK11	hsa-miR-339-5p	0.037138	up	16.63	MIMAT0000764	NO
STK11	hsa-miR-34a-3p	0.047655	up	50.77	MIMAT0004557	NO
STK11	hsa-miR-4252	0.014764	up	55.13	MIMAT0016886	NO
STK11	hsa-miR-4318	0.001537	up	54.74	MIMAT0016869	NO
STK11	hsa-miR-4328	0.035986	up	22.42	MIMAT0016926	NO
STK11	hsa-miR-4730	0.03915	down	-45.98	MIMAT0019852	NO
STK11	hsa-miR-4770	0.010971	up	39.62	MIMAT0019924	NO
STK11	hsa-miR-489-3p	0.004512	up	43.13	MIMAT0002805	NO
STK11	hsa-miR-517a-3p	0.019567	up	22.36	MIMAT0002852	NO
STK11	hsa-miR-517c-3p	0.021522	up	21.18	MIMAT0002866	NO
STK11	hsa-miR-522-3p	0.026955	up	6.25	MIMAT0002868	NO
STK11	hsa-miR-548aa	0.048199	up	13.60	MIMAT0018447	YES
STK11	hsa-miR-5701	0.035075	up	35.87	MIMAT0022494	NO
STK11	hsa-miR-585-3p	0.024521	up	4.84	MIMAT0003250	NO
STK11	hsa-miR-6073	0.026801	up	36.71	MIMAT0023698	NO
STK11	hsa-miR-624-5p	0.009898	up	32.12	MIMAT0003293	NO
STK11	hsa-miR-628-5p	0.032169	up	27.20	MIMAT0004809	NO
STK11	hsa-miR-6516-3p	0.047177	up	34.70	MIMAT0030418	NO
STK11	hsa-miR-6872-3p	0.016967	up	8.28	MIMAT0027645	NO
STK11	hsa-miR-8077	8.18E-04	up	115.11	MIMAT0031004	NO
TP53	hsa-miR-147b	0.023046	up	3.07	MIMAT0004928	NO
TP53	hsa-miR-205-3p	0.018824	up	11.09	MIMAT0009197	YES
TP53	hsa-miR-4290	0.017774	up	6.99	MIMAT0016921	NO
TP53	hsa-miR-6891-3p	0.043351	up	4.85	MIMAT0027683	NO

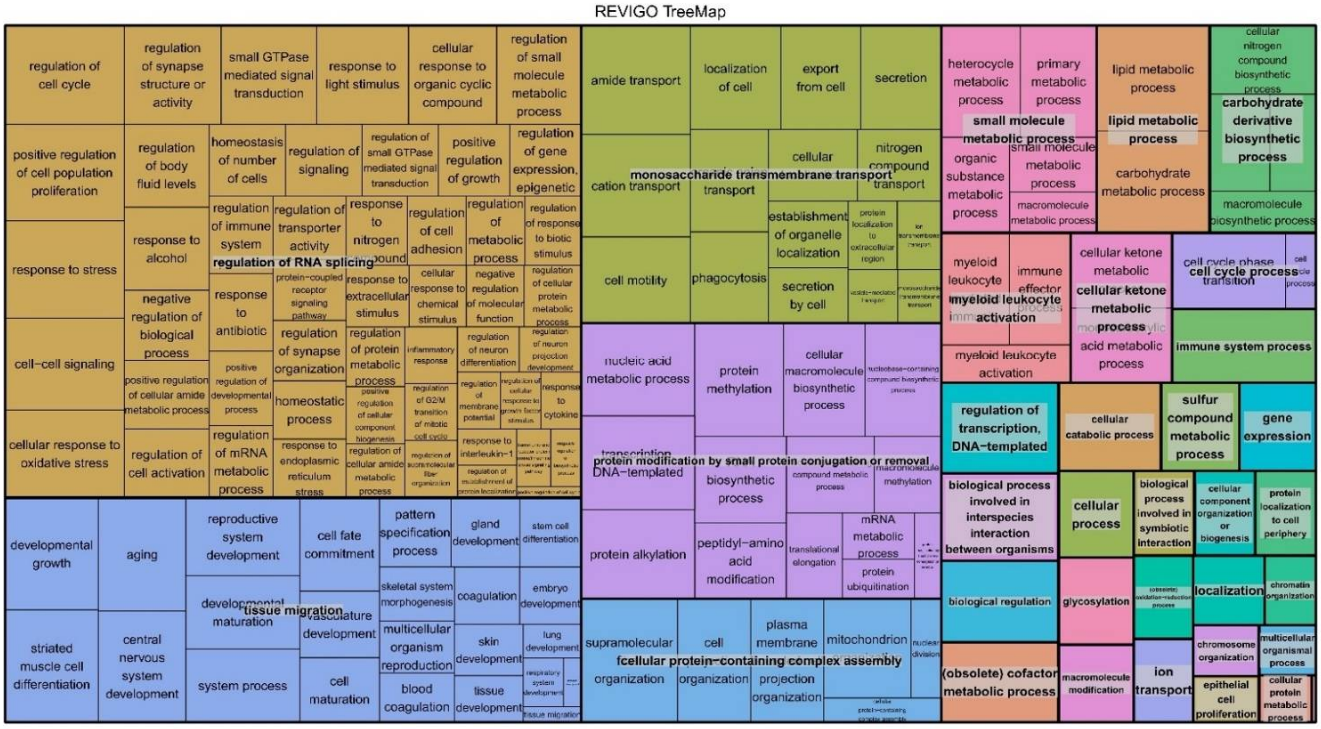

Figure S1. Revigo TreeMap of GO-BP analysis. Most significative BPs are classified by colour with BPs subset.
